# Effect of Bile on Hemodynamics and Blood Micro-Rheological Parameters in Experimental Models of Bilhemia

**DOI:** 10.3390/metabo14040211

**Published:** 2024-04-07

**Authors:** Adam Attila Matrai, Adam Varga, Laszlo Adam Fazekas, Barbara Bedocs-Barath, Noel Johny Nellamkuzhi, Tran Bao Nghi, Norbert Nemeth, Adam Deak

**Affiliations:** Department of Operative Techniques and Surgical Research, Faculty of Medicine, University of Debrecen, Moricz Zsigmond u. 22, H-4032 Debrecen, Hungary; matrai.adam@med.unideb.hu (A.A.M.); varga.adam@med.unideb.hu (A.V.); fazekas.laszlo@med.unideb.hu (L.A.F.); barath.barbara@med.unideb.hu (B.B.-B.); evzksd@mailbox.unideb.hu (N.J.N.); meimei@mailbox.unideb.hu (T.B.N.); deak.adam@med.unideb.hu (A.D.)

**Keywords:** bilhemia, hemodynamics, hemorheology, red blood cell deformability, red blood cell aggregation

## Abstract

As a rare complication of liver injury and certain interventions, bile can enter the bloodstream depending on the pressure gradient, resulting in bilhemia. Its micro-rheological and hemodynamic effects are still unclear. We aimed to study these parameters in experimental bilhemia models. Under general anesthesia, via laparotomy, bile was obtained by gallbladder puncture from pigs and by choledochal duct cannulation from rats. In vitro, 1 µL and 5 µL of bile were mixed with 500 µL of anticoagulated autologous blood. The systemic effect was also assessed (i.v. bile, 200 µL/bwkg). Hemodynamic and hematological parameters were monitored, and red blood cell (RBC) deformability and aggregation were determined. RBC deformability significantly decreased with the increasing bile concentration in vitro (1 µL: *p* = 0.033; 5 µL: *p* < 0.001) in both species. The RBC aggregation index values were concomitantly worsened (1 µL: *p* < 0.001; 5 µL: *p* < 0.001). The mean arterial pressure and heart rate decreased by 15.2 ± 6.9% and 4.6 ± 2.1% in rats (in 10.6 ± 2.6 s) and by 32.1 ± 14% and 25.2 ± 11.63% in pigs (in 48.3 ± 18.9 s). Restoration of the values was observed in 45 ± 9.5 s (rats) and 130 ± 20 s (pigs). Bilhemia directly affected the hemodynamic parameters and caused micro-rheological deterioration. The magnitude and dynamics of the changes were different for the two species.

## 1. Introduction

Bilhemia is an uncommon condition that occurs when bile enters the bloodstream due to a bilio-venous fistula between the bile duct and the hepatic venous system [1]. This can lead to various complications. Bilhemia is not a common condition, but its exact prevalence is not known, as milder forms of the condition are unlikely to be recognized, diagnosed, or recorded, and there is no information on the mortality rates. Bilhemia can be a rare side effect of bile duct stones [2], and the typical common bile duct pressure, which ranges from 10–15 to 20 mmHg (when the sphincter of Oddi contracts), is involved in its pathogenesis. Bilhemia was first documented in 1559, and about 100 instances have been described since then, with bradycardia and a decrease in blood pressure. It is important to differentiate bilhemia from hemobilia, which is bleeding into the biliary tract caused by a fistula connecting the bile duct to the hepatic blood vessels [2,3,4,5,6,7]. Because of the high pressure within the arterial system, in normal circumstances bilhemia is not possible in the presence of an arterial-biliary fistula [2,8,9,10].

The most frequent cause of bilio-venous fistulas (BVFs) is severe liver trauma [2,11]. A rare side effect of percutaneous transhepatic biliary drainage is likewise a BVF [12]. Furthermore, a BVF is an uncommon but dangerous side effect of blunt liver injuries in children [1]. Additionally, a case report details how a cholecystectomy resulted in the formation of a BVF, which ultimately caused death [13,14]. Bilhemia can be associated with bile duct stones, which can cause abdominal pain [3].

Many hemorheological abnormalities have been noted in icterus (jaundice) patients. These alterations include a reduction in the deformability of red blood cells, an increase in erythrocyte aggregation, and a compromise of blood viscosity. Changes in the oxidant–antioxidant status, endotoxemia, and hyperlipidemia are among the causes linked to the hemorheological effects of icterus [15,16]. Furthermore, research demonstrated that jaundice can impact erythrocytes and change the rheology of blood, which may cause diseases such liver failure, hepatic encephalopathy, and portal hypertension [17]. However, there are no data about the hemorheological effects of bilhemia.

Bile acids or their conjugate bile salts build up in the liver as a result of the obstruction of the biliary ducts. Although they have been thoroughly investigated, the molecular mechanisms behind the liver damage linked to cholestasis remain poorly understood. A notable inflammatory infiltration is observed in the areas of necrosis that define various forms of obstructive cholestasis [18]. Cholestasis can affect blood viscosity [15,19]. Bilirubin and bile acids may affect erythrocytes, leading to changes in blood viscosity [15]. Cholestasis is associated with an increase in erythrocyte aggregation [19]. This is attributed to factors such as hyperlipidemia, endotoxemia, and changes in the oxidant–antioxidant status [19,20,21,22]. It can also affect the deformability of erythrocytes, which is another important parameter in hemorheology [23]. The impairment of blood rheology by cholestatic jaundice in humans has also been investigated, and it was found that bilirubin and bile acids may affect erythrocytes [15].

Bilhemia is most easily diagnosed by the methods used for hemobilia examination [24]. Interestingly, abdominal computed tomography (CT) is a less sensitive method for identifying biliary bleeding. Magnetic resonance cholangiography or transabdominal ultrasonography may be more sensitive in this regard. Another method is isotope-labeled red blood cell scanning or endoscopic retrograde cholangiopancreatography (ERCP), which may help to localize the source of bleeding [25,26].

We hypothesized that bilhemia has a direct effect on red blood cell conventional and osmotic gradient deformability and red blood cell aggregation, and the dynamics of these changes together with hemodynamic alterations may show inter-species differences as well, since hemodynamics [27,28], bile composition [29,30,31], and hemorheological parameters differ in species [32,33] and various diseases [34,35,36,37]. This pilot study aimed to investigate the in vitro and in vivo direct effect of bile on blood micro-rheological parameters—such as red blood cell deformability and aggregation—heart rate, and mean arterial pressure in vivo, in rats and pigs.

## 2. Materials and Methods

### 2.1. Experimental Animals and Sampling Protocol

The experiments were carried following the European Union Directive and National Regulations and with the approval of the University of Debrecen Committee of Animal Welfare (reg. Nr.: 17/2022/UDCAW). Six healthy male Wistar (Crl:WI) rats (bodyweight: 458.3 ± 24.5 g) were used at 12–14 months of age in this study. The rats were kept in standard cages (Eurostandard IV, Tecniplast, Buguggiate, Italy) at a temperature of 22 ± 2 °C, humidity of 55% ± 10%, lighting on a 12–12 h light/dark cycle, and with free access to water and standard rat food. Also, we included six female Hypor pigs (12–13-week-old, bodyweight: 20.8 ± 1.7 kg) in this study. Ventilation (15–20× air change/hour) and heating (central and underfloor heating) of the enclosures were provided, in a temperature range of 22–26 °C, according to the weight of the animals. Extreme and sudden wide fluctuations in humidity were avoided during the housing of the animals. The animals were supplied with a feed mix appropriate for their species, and water was provided by a self-watering system.

#### 2.1.1. Experimental Protocol in Rats

Under general anesthesia with 100 mg/bwkg of ketamine i.p. (CP-ketamine hydrochloride 10%, Produlab Pharma BV, Raamsdonksveer, The Netherlands), 10 mg/bwkg of xylazine i.p. (CP-xylazine hydrochloride, 2%; Produlab Pharma BV, Raamsdonksveer, The Netherlands), a 26 G cannula was inserted into the lateral tail vein for blood sampling, fluid therapy, and bile administration. The right common carotid artery was prepared and cannulated (O.D. 0.965 mm, Polyethylene Tubing Clay Adams, 427411, BD Intramedic^TM^, Sollentuna, Sweden). After fixation with a central ligature, the cannula was connected to an invasive hemodynamic monitoring system (Hemosys monitor system LD-01, Experimetria Ltd., Budapest, Hungary). For bile collection, median laparotomy was performed, and the ductus choledochus was prepared, opened by microsurgical techniques, and cannulated (Micro-Renathane^®^, MRE-025 type. 0.25 mm outer diameter, × 0.12 mm inner diameter; Braintree Sceintific Inc., Braintree, MA, USA) [38,39]. A 1 mL syringe was connected to the cannula with a 27 G needle. Bile was collected for 1 h, providing an extracted bile volume of approximately 250 µL.

#### 2.1.2. Experimental Protocol in Pigs

The following anesthesia protocol was used: for pre-medication, i.m. 1–2 mg/kg of azaperone (Stresnil, Elanco GmbH, Cuxhaven, Germany); for induction of anesthesia, i.m. 2 mg/kg of xylazine (CP-xylazine hydrochloride, 2%) and 20 mg/kg of ketamine (CP-ketamine hydrochloride 10%); for maintenance of permanent anesthesia, i.v. 1 mg/kg of xylazine and 10 mg/kg of ketamine, supplemented with i.v. 2 mg/kg of diazepam (Diazepeks 5 mg/ml, AS Grindeks, Riga, Latvia). The animals were intubated, and external jugular vein cannulation (Certofix Trio, 7F, B.Braun Trading Ltd., Budapest, Hungary) was performed unilaterally for blood sampling, fluid therapy, and bile administration. The left common carotid artery was also cannulated (Certofix Trio, 7F) and connected to an invasive hemodynamic system (Hemosys monitor system LD-01). Bile was obtained by direct puncture of the gallbladder via upper median laparotomy. For this, 21 G needles and a syringe with a volume of 5 mL were used, and 5 mL of bile was collected.

#### 2.1.3. Blood Sampling Protocol

Before surgery and bile administration, venous blood (for in vitro studies, 1.5 mL, for in vivo studies, 0.5 mL of sodium EDTA 1.8 mg/mL) was collected from the cannulated veins. Baseline measurements were performed, and the effects of bile in vitro were investigated after adding 1 µL or 5 µL of bile to 500 µL of blood. In the in vivo studies, blood samples were taken 5 min after i.v. bolus injection of 200 µL/bwkg of bile.

### 2.2. Hematological Parameters

Red blood cell count (RBC [T/L]), white blood cell count (WBC [G/L]), hemoglobin concentration (Hgb [g/dL]), hematocrit (Hct [%]), mean corpuscular volume (MCV [fL]), mean corpuscular hemoglobin (MCH [pg]), mean corpuscular hemoglobin concentration (MCHC [g/dL]), and platelet count (Plt [G/L]) were measured using a Sysmex K-4500 microcell counter (TOA Medical Electronics Co., Ltd., Kobe, Japan).

### 2.3. Red Blood Cell Conventional and Osmotic Gradient Deformability Measurements

A LoRRca Maxsis Osmoscan ektacytometer (RR Mechatronics International B.V., Zwaag, The Netherlands) was used to determine the deformability of red blood cells [40,41]. The blood samples were sheared, and laser diffraction techniques were used to measure the elongation of the cells. The so-called elongation index (EI) was determined as a function of shear stress (SS, Pa; range: 0.3 to 30 Pa). In order to perform the traditional deformability test, 2 mL of a polyvinylpyrrolidone (PVP)–PBS solution (PVP: 360 kDa, Sigma-Aldrich Co., St. Louis, MO, USA; PVP-PBS solution viscosity = 30.5 mPas, osmolality = 303 mOsmol/kg, pH = 7.5) was carefully mixed with 10 μL of sample (whole blood or blood–bile suspension). Every measurement was performed at 37 °C [30]. Comparative data from the EI–SS curves were calculated, i.e., EI values at 3 Pa, and the Lineweaver–Burk equation (1/EI = SS_1/2_/EI_max_ × 1/SS + 1/EI_max_) was used for the parameterization of individual EI–SS curves, providing the maximal elongation index (EI_max_) and the shear stress at half EI_max_ (SS_1/2_, Pa) [42]. Lowe EI or EI_max_ and high SS_1/2_ values represent impaired red blood cell deformability [43].

Measurements of osmotic gradient deformability (osmoscan) were performed with 250 μL of sample and 5 mL of isotonic PVP-PBS (see above). As the device mixed low-osmolarity (0 mOsm/kg) and high-osmolarity (500 mOsm/kg) PVP solutions with the sample, the osmolality of the suspension varied. In this module, the determination of EI was carried out at constant shear stress (30 Pa). As the osmolality of the blood sample was steadily increased, the sample was aspirated into this PVP solution, and the elongation index was continually recorded. The outcome was a recognizable EI–osmolality (O) curve with multiple noteworthy spots, such as, in a low-osmolality range, the minimal elongation index (EI min) and the associated osmolality value (O min), as well as the maximal elongation index (EI max, note that it is not the same as EI_max_ in the Lineweaver–Burk equation, see above) and the associated O (EI max) value, and in the higher osmolality range, EI hyper (half of the maximal elongation index in the high-osmolarity environment) and O hyper. The area under each unique EI–O curve was calculated [33,40]. Additional parameters, such as ΔEI (absolute difference between maximal and minimal EI values), ΔO (absolute difference between osmolality values at maximal and minimal EI), and ratio values, such as EI max/EI min (rEI), O (EI max)/O min (rO), ΔEI/ΔO, and rEI/rO, were also calculated [33].

### 2.4. Determination of Red Blood Cell Aggregation

A Myrenne MA-1 erythrocyte aggregometer (Myrenne GmbH, Roetgen, Germany) was used to determine the aggregation index values of the blood samples. The technique is based on the light transmittance photometric method. The test requires 20 µL of blood. After disaggregation by a controlled shearing system (shear rate: 600 s^−1^), light transmission was tested for 5 or 10 s at stasis (M values, shear rate: 0 s^−1^) or at a low shear (M1 values, shear rate: 3 s^−1^) [44,45]. The measurements were carried out at room temperature (20–25 °C). High index values (M 5 s, M1 5 s, M 10 s, M1 10 s) represent enhanced RBC aggregation [40,44,46,47,48,49].

### 2.5. Statistical Analysis

To estimate the necessary sample number (sample size) for the experiment, Mead’s resource equation method was used. For statistical analyses, SigmaStat Software 3.1.1.0. was used (Systat Software Inc., San Jose, CA, USA). For general data presentation, means ± S.D. (standard deviation) are shown. After testing the normality of the data distribution by the Kolmogorov–Smirnov test, differences between the doses were analyzed by *t*-test or the Mann–Whitney rank sum test, and one-way ANOVA or Kruskal–Wallis’s test was used based on the results of the normality test. A *p*-value of < 0.05 was considered statistically significant.

## 3. Results

### 3.1. Hemodynamic Variables

**Figure 1:**
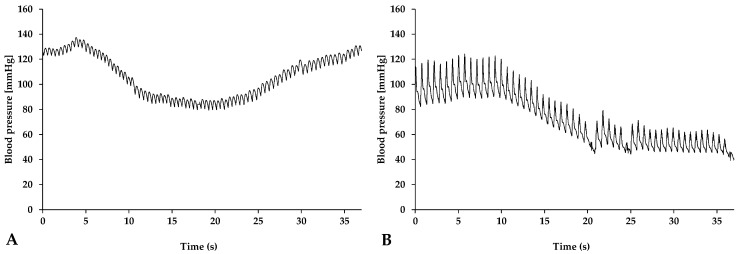
Representative curves of mean arterial pressure after bile administration in rats (**A**) and pigs (**B**).

Figure 1 shows representative curves of the mean arterial pressure changes after bile injection in both species.

After bile injection, the mean arterial pressure started to decrease rapidly. In rats, the values were normalized within a short period of time, while in pigs, their restoration was much slower (Table 1).

### 3.2. Hematological Parameters

Table 2 provides a summary of the general quantitative and qualitative hematological parameters.

For rats, a slight increase was observed in the examined parameters, except for the white blood cell count, which was significant for hemoglobin (*p* = 0.002 vs. 1 µL of bile; *p* = 0.015 vs. 5 µL of bile; *p* = 0.048 vs. in vivo 200 µL/bwkg of bile) and hematocrit values (*p* < 0.001 vs. 1 µL of bile; *p* = 0.006 vs. 5 µL of bile; *p* < 0.001 vs. in vivo 200 µL/bwkg of bile), compared to intact whole blood. In contrast, for pigs, a slight decrease or stagnation was observed for most parameters, compared to intact blood.

### 3.3. Red Blood Cell Deformability

When the elongation index–shear stress curves were evaluated, different variation was observed between species. For rats, after both in vitro and in vivo bile administration, a decrease and then an increase in the elongation index values were observed in the low shear stress range (<3 Pa) and normalization in the high shear stress range (>3 Pa), compared to intact whole blood. For pigs, a decrease in the elongation index values was observed following the in vitro administration of 1 µL of bile, whereas an increase in the EI values at low shear stress (<3 Pa) and a decrease at high shear stress (>3 Pa) were observed following the in vitro administration of 5 µL and the in vivo administration of 200 µL/bwkg of bile, respectively, compared to intact whole blood.

**Figure 2:**
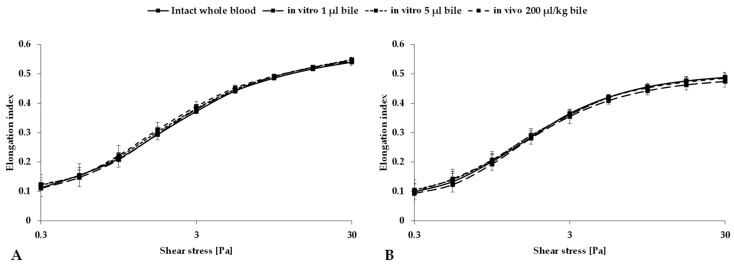
Elongation index as a function of shear stress (SS [Pa]) for rats (**A**) and pigs (**B**), for mixtures of bile and blood (in vitro, 1 µL or 5 µL of bile in 500 µL blood) and for samples obtained after the induction of bilhemia (in vivo, 200 µL/bwkg), compared to base values (intact blood). Means ± S.D.

Figure 2 illustrates the elongation index (EI) as a function of shear stress (SS); the numerical data of the conventional and osmotic gradient deformability tests are summarized in Table 3 and Table 4.

### 3.4. Red Blood Cell Aggregation

**Figure 3:**
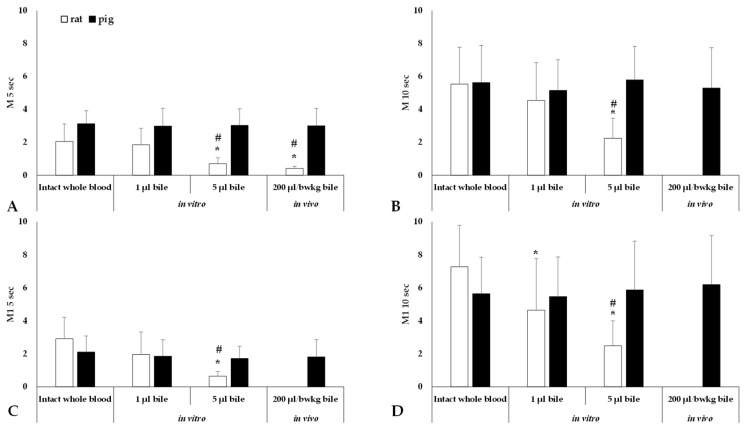
Aggregation index values measured in stasis for 5 or 10 s (M 5 s, M 10 s) and at a shear rate of 3 s^−1^ for 5 or 10 s (M1 5 s, M1 10 s) after disaggregation. Mean ± S.D.; * *p* < 0.05 vs. intact blood; # vs. 1 µL bile.

Figure 3 presents the alterations in red blood cell aggregation indices.

For the rat samples, comparing the two in vitro and an in vivo doses, in vitro, 5 µL of bile significantly decreased the aggregation index values compared to that of intact whole blood (*p* < 0.001), and 1 µL of bile (M 5 s, M1 5 s, and M 10 s: *p* < 0.001; M1 10 s: *p* = 0.017) affected all four parameters. Following the administration of 200 µL/bwkg of bile in vivo, significant differences in the aggregation index values were observed between the groups when measured under static conditions after 5 s (M 5 s) of disaggregation (*p* = 0.002 vs. base conditions, *p* = 0.003 vs. 1 µL of bile). The values of the other three parameters (M1 5 s, M 10 s, M1 10 s) were extremely low.

For pigs, no significant changes in the aggregation index values were observed when comparing intact whole blood with samples after in vitro or in vivo treatment with bile, in contrast to the significant differences observed in rats (Figure 3).

## 4. Discussion

In our study, we found that the mean arterial pressure decreased in both species after intravenous bile administration. In rats, all hematological parameters, except white blood cell count, showed an increase in vitro and in vivo, while in pigs, a slight increase or stagnation was observed. No significant changes in red blood cell conventional and osmotic gradient deformability were observed. A significant decrease in red blood cell aggregation was observed in rat samples after the in vitro administration of 5 µL of bile, with an unmeasurable decrease in aggregation after in vivo bile administration. No significant difference in the aggregation index values was observed in pigs.

The calculation of the bilhemia dosage was based on the in vitro dose of 1 µL. Accordingly, 1 µL of bile was added to 500 µL whole blood, resulting in a bile–blood suspension of 0.2%. The average blood volume in rats is 54–70 mL/bwkg, while that in pigs is 56–69 mL/bwkg [50]. So, the circulating blood volume in rats ranges from 25 to 32 mL, and that in pigs from 1165 to 1435 mL. Following the administration of 200 µL/bwkg, a bile-to-blood ratio of approximately 0.2–0.3% was achieved in vivo.

Bile acids affect blood pressure and heart rate [30,51]. Previous studies demonstrated that heart disease can result from aberrant bile acid metabolism [52,53,54,55]. Heart rate variability, stress response sensitivity, and QT interval lengthening are linked to conditions such as primary biliary cholangitis and intrahepatic cholestasis of pregnancy, which can raise the risk of cardiovascular complications [56,57,58]. Furthermore, cirrhotic cardiomyopathy, a frequent consequence of liver cirrhosis marked by extended QT intervals and systolic or diastolic failure, might be linked to bile acids’ impact on cardiovascular function [59,60].

Though there is a great deal of disagreement, three theories have been put up to explain how bile acids could cause bradycardia [61]: (1) by creating a monolayer on the surface of the cell membrane to cause mechanical interference [62]; (2) by lowering the sluggish inward calcium current, preventing the membrane from conducting action potentials [63]; (3) by functioning as an antimuscarinic antagonist, since physostigmine can intensify or counteract their effects [62].

Moreover, gallbladder inflammation can trigger nerve reflexes that lower blood pressure and heart rate and possibly cause cardiac ischemia or arrest [57]. Heart rate and arterial blood pressure can be impacted by reflex coronary vasoconstriction, which is brought on by the connection between gallbladder distension and variations in coronary blood flow [64].

It was demonstrated that bile acids cause a process known as eryptosis, i.e., cell death of erythrocytes [65]. The process by which bile acids cause red blood cells to absorb more calcium is mediated by the activation of cation channels. This raises cytosolic Ca^2+^ activity and ultimately causes eryptosis, the suicidal death of erythrocytes marked by the exposure of phosphatidylserine on the cell surface [65,66]. It was demonstrated that bile salt-associated hemolysis is partially calcium-mediated [66,67]. Moreover, the bile acid concentrations needed to induce eryptosis are greater than those needed to cause hemolysis [65].

Exercise was also shown to influence alterations in human bile and red blood cell lipids [54]. Moreover, increased red blood cell distribution width was linked to pregnancy-related intrahepatic cholestasis, a disorder that impairs the bile flow [68].

Results indicate that the red blood cell uptake of calcium is influenced by bile acid concentration. Human erythrocytes were demonstrated to absorb calcium in vitro when exposed to bile salts, and the amount of calcium absorbed depends on bile salts’ concentration [66]. Bile salts cause hemolysis at high doses by co-micellizing the lipid components of the cell membrane [66]. However, through as-yet-undefined mechanisms, bile salts are also linked to hemolysis at lower concentrations [66]. Calcium uptake is stimulated 4- to 25-fold when bile salts are present, and the amount of calcium absorbed depends on the bile salt concentration [66]. The degree of this increase was attributed to ATP depletion or exposure to trifluoperazine, both of which reduce red blood cell calcium pump activity [66]. Furthermore, calcium increases the hemolytic activity of bile salts; this effect is greatest in a buffer containing 100 mM KCl/50 mM of NaCl [62]. In conclusion, the concentration of bile salts determines the amount of calcium uptake in red blood cells, which is influenced by the concentration of bile acids.

Bile acids can also stimulate the intake of sodium and/or the export of intracellular potassium into erythrocytes, which can result in lysis [69]. Moreover, ceramide and Ca^2+^ entry have a similar impact to that of bile acids in stimulating suicidal cell death [65].

There are notable variations in the composition of bile acids among rats, pigs, and humans when their bile chemistry is compared. Studies showed that bile samples from rats and pigs have significant quantitative differences in the amounts of various bile acids, including taurocholic acid (TCA) and glycocholic acid (GCA), with changes of more than 400% in certain instances [29]. Furthermore, research demonstrated that there are significant differences in the composition of plasma, urine, and bile acids, as well as their metabolites, between various species, with minipigs, rats, and mice displaying the most divergent bile acid profiles in comparison to humans [30]. Additionally, a possible connection between the composition of bile acids and the activity of calcium ionophores in the intestine was suggested by an investigation conducted on pigs concerning the relationship between bile acids’ reported intestinal fluid secretory activity and their properties [55].

A variety of factors may be involved in the variations in bile acid composition found in different animals. These variables include variations in the enterohepatic circulation of bile acids, intestinal absorption, and liver metabolism [70,71]. Furthermore, various species differ in the expression and activity of bile acid transporters as well as of the enzymes involved in the synthesis and metabolism of bile acids [71,72]. Calcium transport and ionophore activity are two physiological and pharmacological processes that may be affected by species-specific variations in bile acid composition [29]. In conclusion, a variety of complex and multifactorial variables, including variations in intestinal absorption, enterohepatic circulation, liver metabolism, and the expression and activity of bile acid transporters and enzymes, contribute to interspecies differences in bile acid composition and thus to differences in hemorheology and microcirculation due to bilhemia.

Limitations of our study include the fact that only two animal species were studied. We only tested two doses in vitro and used a short incubation period in our in vivo experiments, so that only acute changes could be detected. Another limitation of the present research is that there is anatomical difference between swine and rats regarding the presence or absence of the gallbladder. Pigs have, like humans, a well-defined gallbladder, but in rats, this anatomical structure is missing. This fact influenced the method of bile sampling: from rats bile was collected from the cannulated ductus choledochus, while from pigs it was obtained directly from the cholecyst.

## 5. Conclusions

It is concluded that bilhemia directly affects hemodynamic parameters and causes micro-rheological deterioration. The mean arterial pressure and heart rate decreased in both species in vivo. No changes in parameters describing red blood cell deformability were observed after bile administration. There was a significant difference in the aggregation index values between the two examined species. The red blood cells of rats were more sensitive after both in vitro and in vivo bile administration. In vitro, a possible dose-dependent effect was also observed. The magnitude and dynamics of the acute hemodynamic effects of bilhemia were different for the two species studied. The observed micro-rheological and microcirculatory changes may be part of the complications of liver injury through the direct action of bile and bile acids.

Further studies are needed to better understand the effect of bile on red blood cells. Potential studies could include an increased number of animals, with the usage of different species, doses, and administration frequency, to investigate the possible long-term effects of bilhemia. Supplementary research could be performed to elucidate the assumptions made in this study, improve the diagnosis of bilhemia, and develop potential new therapies for clinical practice.

## Figures and Tables

**Table 1:** Rate of decrease in mean arterial pressure (MAP) and heart rate (HR), with duration of the decrease and time required for normalization.

Species		Rat		Pig	
Parameter		MAP	HR	MAP	HR
rate of decrease (%)		15.2 ± 6.9	4.6 ± 2.1	32.1 ± 14 *	25.2 ± 11.6 *
duration of decrease (s)		10.6 ± 2.6		48.3 ± 18.9 *	
time required for normalization (s)	50%	24.4 ± 7		83.3 ± 25.2 *	
	80%	34.8 ± 8.4		110.3 ± 22 *	
	100%	45 ± 9.5		130 ± 20 *	

MAP: mean arterial pressure, HR: heart rate; means ± S.D.; * *p* < 0.05 vs. rat.

**Table 2:** Alterations of hematological parameters of mixtures of bile and blood (in vitro, 1 µL or 5 µL of bile in 500 µL blood) and for samples obtained after the induction of bilhemia (in vivo, 200 µL/bwkg), compared to base values (intact blood samples).

	Intact Blood		In Vitro 1 µL Bile		In Vitro 5 µL Bile		In Vivo (Bilhemia)	
	Rat	Pig	Rat	Pig	Rat	Pig	Rat	Pig
RBC [10^12^/L]	8.25 ± 0.33	6.95 ± 0.64	8.76 ± 0.58	6.42 ± 0.71 *	8.6 ± 0.43	6.52 ± 0.65	8.68 ± 0.5	7.01 ± 1.18
WBC [10^9^/L]	7.08 ± 1.31	24.78 ± 2.74	6.98 ± 2.48	25.01 ± 2.96	6.63 ± 1.93	24.57 ± 2.75	6.71 ± 1.92	21.78 ± 4.3
Hgb [g/L]	147.7 ± 5.5	123.8 ± 14.9	159 ± 8.5 *	115.1 ± 13.4	156.4 ± 7.2 *	116.9 ± 12.6	160.6 ± 7.8 *	123.9 ± 19.9
Hct [%]	44.35 ± 1.33	41.09 ± 4.67	48.34 ± 2.61 *	38.09 ± 4.22	47.57 ± 2.19 *	38.76 ± 3.95	48.64 ± 2.65 *	40.86 ± 6.91
MCV [fL]	53.82 ± 1.33	59.04 ± 2.26	55.24 ± 1.76	59.33 ± 1.92	55.36 ± 1.67	59.48 ± 2.01	56.03 ± 1.74 *	59.07 ± 2.06
MCH [pg]	17.31 ± 1.9	17.79 ± 0.78	18.19 ± 1.01	17.93 ± 0.92	18.21 ± 0.71	17.93 ± 0.93	18.51 ± 0.69	17.88 ± 0.93
MCHC [g/L]	321.5 ± 32.8	301.2 ± 4.9	329 ± 10.5	302 ± 7	328.8 ± 3.7	301.4 ± 6.8	330.2 ± 4.4	302.9 ± 8.1
Plt [10^9^/L]	744.6 ± 175.1	409.9 ± 94.3	808.5 ± 114.9	441.9 ± 78.8	785.8 ± 110.5	427.6 ± 66.5	862.7 ± 148.9	385.6 ± 110.7

RBC: red blood cell count; WBC: white blood cell count; Hgb: hemoglobin; Hct: hematocrit; MCV: mean corpuscular volume; MCH: mean corpuscular hemoglobin; MCHC: mean corpuscular hemoglobin concentration; Plt: platelet count. Means ± S.D.; * *p* < 0.05 vs. intact blood.

**Table 3:** Alterations in red blood cell deformability parameters for mixtures of bile and blood (in vitro, 1 µL or 5 µL of bile in 500 µL blood) and for samples obtained after the induction of bilhemia (in vivo, 200 µL/bwkg), compared to base values (intact blood samples).

	Intact Blood		In Vitro 1 µL Bile		In Vitro 5 µL Bile		In Vivo (Bilhemia)	
	Rat	Pig	Rat	Pig	Rat	Pig	Rat	Pig
EI at 3 Pa	0.372 ± 0.01	0.365 ± 0.01	0.379 ± 0.02	0.355 ± 0.02	0.381 ± 0.01	0.361 ± 0.02	0.389 ± 0.02 *	0.36 ± 0.02
EI_max_	0.568 ± 0.01	0.521 ± 0.02	0.574 ± 0.01	0.503 ± 0.02	0.573 ± 0.02	0.517 ± 0.03	0.571 ± 0.02	0.512 ± 0.02
SS_1/2_ [Pa]	1.611 ± 0.21	1.403 ± 0.27	1.609 ± 0.23	1.347 ± 0.26	1.544 ± 0.24	1.355 ± 0.29	1.463 ± 0.34	1.31 ± 0.23
EI_max_/SS_1/2_ [Pa^−1^]	0.358 ± 0.04	0.383 ± 0.07	0.362 ± 0.05	0.385 ± 0.07	0.381 ± 0.05	0.383 ± 0.08	0.404 ± 0.07	0.402 ± 0.06

EI at 3 Pa: elongation index at 3 Pa; EI_max_: calculated maximal elongation index; SS_1/2_: shear stress at half EI_max_. Means ± S.D.; * *p* < 0.05 vs. intact blood.

**Table 4:** Alterations in red blood cell osmotic gradient deformability (osmoscan) parameters for mixtures of bile and blood (in vitro, 1 µL or 5 µL of bile in 500 µL blood) and for samples obtained after the induction of bilhemia (in vivo, 200 µL/bwkg), compared to base values (intact blood samples).

	Intact Blood		In Vitro 1 µL Bile		In Vitro 5 µL Bile		In Vivo (Bilhemia)	
	Rat	Pig	Rat	Pig	Rat	Pig	Rat	Pig
EI min	0.156 ± 0.012	0.147 ± 0.013	0.154 ± 0.007	0.142 ± 0.014	0.159 ± 0.016	0.139 ± 0.006	0.158 ± 0.014	0.145 ± 0.008
EI max	0.525 ± 0.023	0.528 ± 0.005	0.532 ± 0.024	0.528 ± 0.004	0.528 ± 0.019	0.528 ± 0.004	0.532 ± 0.03	0.521 ± 0.008
EI hyper	0.263 ± 0.012	0.264 ± 0.003	0.266 ± 0.012	0.264 ± 0.002	0.264 ± 0.01	0.264 ± 0.002	0.266 ± 0.015	0.261 ± 0.004
O min [mOsm/kg]	136 ± 6.03	182 ± 7.86	137.17 ± 7.17	182 ± 5.76	139.33 ± 6.92	179 ± 4.69	142.67 ± 5.68	183 ± 8.16
O (EI max) [mOsm/kg]	277 ± 17.4	357.88 ± 10.09	281.67 ± 21.84	354.13 ± 6.56	280.33 ± 26.11	355.43 ± 9.69	288.17 ± 16.14	352.25 ± 10.86
O hyper [mOsm/kg]	428.33 ± 3.14	492.83 ± 9.95	431 ± 4.16	487 ± 6.16	435.25 ± 6.24	489.8 ± 8.26	439.5 ± 3.7	491 ± 8.17
Area	141.85 ± 9.56	135.03 ± 3.71	143.17 ± 7.33	134.33 ± 1.82	139.9 ± 7.71	135.1 ± 3.13	141.92 ± 14.12	132.84 ± 4.01
ΔEI	0.369 ± 0.017	0.381 ± 0.016	0.379 ± 0.021	0.386 ± 0.015	0.369 ± 0.009	0.388 ± 0.009	0.374 ± 0.019	0.377 ± 0.015
ΔO	141 ± 12.92	175.88 ± 10.29	144.5 ± 17.76	172.13 ± 5	141 ± 20.83	176.43 ± 6.5	145.5 ± 14.15	169.25 ± 7.2
rEI	3.38 ± 0.18	3.62 ± 0.35	3.46 ± 0.16	3.75 ± 0.37	3.34 ± 0.24	3.79 ± 0.19	3.38 ± 0.17	3.62 ± 0.25
rO	2.04 ± 0.07	1.97 ± 0.08	2.05 ± 0.11	1.95 ± 0.04	2.01 ± 0.12	1.99 ± 0.03	2.02 ± 0.1	1.93 ± 0.06
ΔEI/ΔO	2.6 × 10^−3^ ± 3 × 10^−4^	2.2 × 10^−3^ ± 2 × 10^−4^	2.7 × 10^−3^ ± 4 × 10^−4^	2.2 × 10^−3^ ± 1 × 10^−4^	2.7 × 10^−3^ ± 4 × 10^−4^	2.2 × 10^−3^ ± 9 × 10^−5^	2.6 × 10^−3^ ± 3 × 10^−4^	2.2 × 10^−3^ ± 1 × 10^−4^
rEI/rO	1.66 ± 0.11	1.84 ± 0.19	1.69 ± 0.14	1.93 ± 0.19	1.66 ± 0.06	1.91 ± 0.1	1.68 ± 0.14	1.88 ± 0.13

EI min: minimal elongation index; O min: osmolality at EI min; EI max: maximal elongation index; O EI max: osmolality at EI max; O hyper: osmolality in the hyperosmolar region at 50% of EI max; EI hyper: half EI max in the hyperosmolar region; ΔEI: absolute difference in EI min and EI max values; ΔO: difference between O min and O (EI max); ratio values: rEI (EI max/EI min), rO (O (EI max)/O min), ΔEI/ΔO, and rEI/rO. Means ± S.D.

## Data Availability

The data presented in this study are available on request from the corresponding author. The data are not publicly available due to ethical permission constraints.
